# Disorders of FZ-CRD; insights towards FZ-CRD folding and therapeutic landscape

**DOI:** 10.1186/s10020-019-0129-7

**Published:** 2019-12-31

**Authors:** Reham M. Milhem, Bassam R. Ali

**Affiliations:** 1 Department of Natural and Applied Sciences, University of Dubai, P.O.Box: 14143, Academic City, Dubai, United Arab Emirates; 2 Department of Pathology, College of Medicine and Health Sciences, United Arab Emirates University, Al-Ain, Abu Dhabi, United Arab Emirates; 3 Zayed Center for Health Sciences, United Arab Emirates University, Al-Ain, Abu Dhabi, United Arab Emirates

**Keywords:** Frizzled cysteine-rich domain, Frizzled receptors, ERAD; protein misfolding, Proteostasis, Lipidation, *cis*-unsaturated fatty acids, Familial exudative vitreoretinopathy; congenital myasthenic syndrome; Robinow syndrome; receptor tyrosine kinase-like orphan receptor 2; frizzled class receptor 4; muscle, Skeletal, Receptor tyrosine kinase; conformational diseases, Cystic fibrosis conductance regulator protein

## Abstract

The ER is hub for protein folding. Proteins that harbor a Frizzled cysteine-rich domain (FZ-CRD) possess 10 conserved cysteine motifs held by a unique disulfide bridge pattern which attains a correct fold in the ER. Little is known about implications of disease-causing missense mutations within FZ-CRD families. Mutations in FZ-CRD of Frizzled class receptor 4 (FZD4) and Muscle, skeletal, receptor tyrosine kinase (MuSK) and Receptor tyrosine kinase-like orphan receptor 2 (ROR2) cause Familial Exudative Vitreoretinopathy (FEVR), Congenital Myasthenic Syndrome (CMS), and Robinow Syndrome (RS) respectively. We highlight reported pathogenic inherited missense mutations in FZ-CRD of FZD4, MuSK and ROR2 which misfold, and traffic abnormally in the ER, with ER-associated degradation (ERAD) as a common pathogenic mechanism for disease. Our review shows that all studied FZ-CRD mutants of RS, FEVR and CMS result in misfolded proteins and/or partially misfolded proteins with an ERAD fate, thus we coin them as “disorders of FZ-CRD”. Abnormal trafficking was demonstrated in 17 of 29 mutants studied; 16 mutants were within and/or surrounding the FZ-CRD with two mutants distant from FZ-CRD. These ER-retained mutants were improperly N-glycosylated confirming ER-localization. FZD4 and MuSK mutants were tagged with polyubiquitin chains confirming targeting for proteasomal degradation. Investigating the cellular and molecular mechanisms of these mutations is important since misfolded protein and ER-targeted therapies are in development. The P344R-MuSK kinase mutant showed around 50% of its in-vitro autophosphorylation activity and P344R-MuSK increased two-fold on proteasome inhibition. M105T-FZD4, C204Y-FZD4, and P344R-MuSK mutants are thermosensitive and therefore, might benefit from extending the investigation to a larger number of chemical chaperones and/or proteasome inhibitors. Nonetheless, FZ-CRD ER-lipidation it less characterized in the literature and recent structural data sheds light on the importance of lipidation in protein glycosylation, proper folding, and ER trafficking. Current treatment strategies in-place for the conformational disease landscape is highlighted. From this review, we envision that disorders of FZ-CRD might be receptive to therapies that target FZ-CRD misfolding, regulation of fatty acids, and/or ER therapies; thus paving the way for a newly explored paradigm to treat different diseases with common defects.

## Background

### Frizzled-like CRD; conserved sequence and structure

**Fig. 1:**
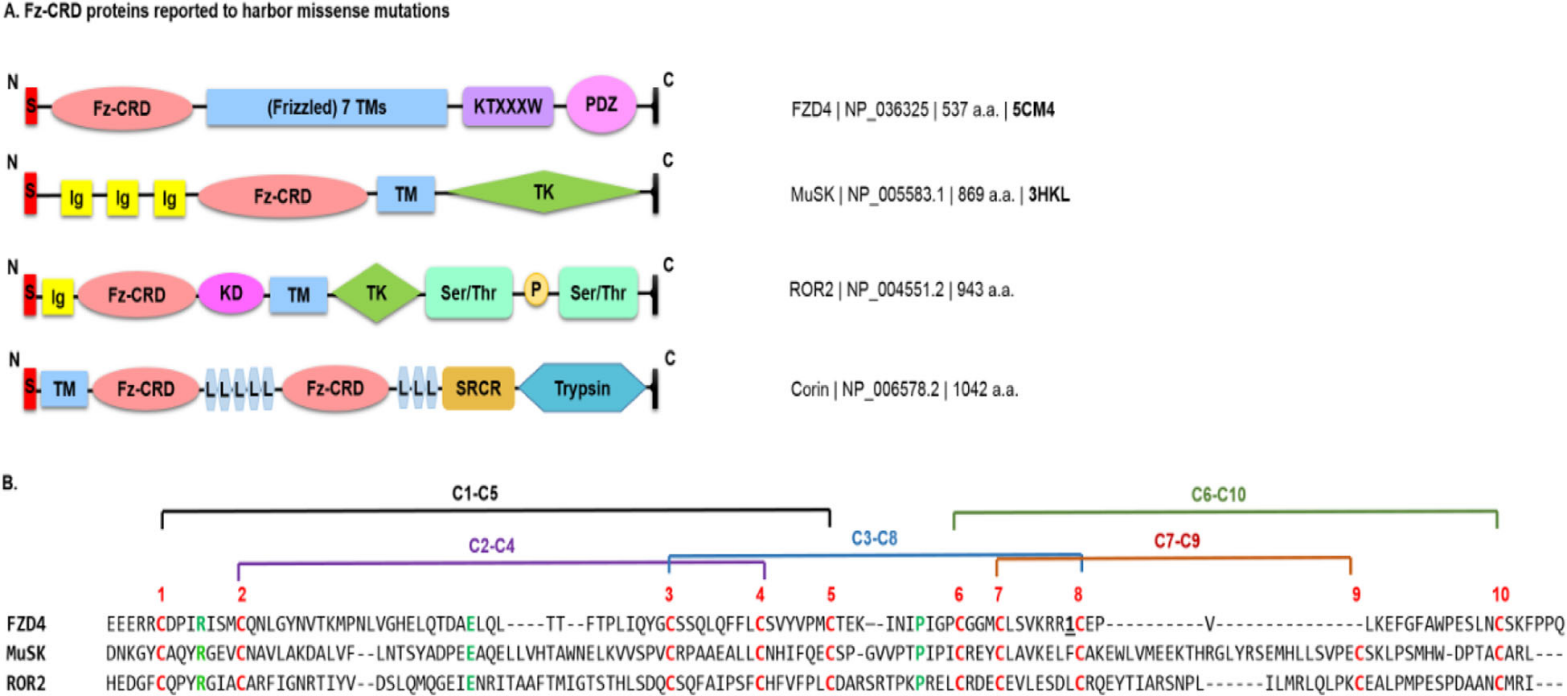
Reported FZD4 proteins with disease-causing missense proteins. **a** Protein domain structural models for FZ-CRD proteins. HUGO gene symbols proteins are shown next to the protein structure and the NCBI accession number is shown next to each protein model. Available PDB codes are in bold at the far right. [PDZ: PDZ binding motif, KTXXXW: lysine-threonine-X-X-X-tryptophan, TM: transmembrane, FZ-CRD: frizzled cysteine- rich domain, TK: tyrosine kinase domain, Ig: immunoglobulin domain, Ser/Thr: Serine-threonine/tyrosine-protein kinase, KD: kinase domain, Trypsin: trypsin-like protease domain, SPCR: scavenger receptor cysteine-rich domain, and L: low density lipoprotein receptor repeats. **b** Multiple sequence alignment of FZD4, MuSK and ROR2 FZ-CRDs. Conserved cysteines are shown in red color. FZ-CRD show homology with a conserved pattern of “CnCnCX8CX6CnCX3CX6,7CnCnC” (Pei and Grishin 2012) *C*: conserved cysteine; *n*: a variable number of residues, *Xn*: n residues, and *Xn1, n2*: n1 to n2 residues in α-helices forming a common Frizzled fold across four α-helices connected by disulfide bridges shown and labelled in red as: “C1–C5, C2–C4, C3–C8, C6–C10, and C7–C9” . For FZD4, one inserted region is shown as the number of inserted residues underlined in bold. Different residues exist between conserved C7 and C8. For FZDs the number is six and RTKs have seven residues

Frizzled receptors (FZD) are G-protein-coupled receptors (GPCRs), which act as gate-keeping proteins, and are receiving considerable attention in recent years. Observing the domains of FZDs shows an amino-terminal (N′) signal peptide (SP) sequence which localizes FZD polypeptides to the endoplasmic reticulum (ER) membrane (Fig. 1a). SP is a hydrophobic rich stretch followed by a cysteine rich region of 120 residues recognized by 10 conserved cysteine motif pattern which is maintained by conserved disulphide bridges holding the α-helical domains of FZD (Fig. 1b). This stitched pattern of cysteine residues by disulfide bridges is known as the Wnt family (Wnt) binding Frizzled cysteine-rich domain (CRD).

Frizzled cysteine-rich domain (CRD), a result of evolutionary membrane fusion, is considered a conserved mobile functional site similar to Frizzled-CRD (FZD-CRD) and is mainly found in Wnt receptors and is shared with other metazoan proteins. The FZD-CRD sequence homology is shared among FZD1–10, and further shares ancestral similarities both in sequence and structure to muscle, skeletal, receptor tyrosine kinase (MuSK); receptor tyrosine kinase-like orphan receptor 2 (ROR2); corin, serine peptidase (CORIN) and the similarity is shown in Fig. 1a. However, other proteins which share the FZD-CRD are smoothened, frizzled class receptor (SMO), secreted frizzled-related proteins (SFRP), carboxypeptidase Z (CPZ); and collagen type XVIII alpha 1 chain (COL18A1), among other proteins (Yan et al. 2013; Saldanha et al. 1998; Pei and Grishin 2012). Henceforth, we refer to the homologous region of FZD-CRD with other proteins as Frizzled-like cysteine-rich domain (FZ-CRD) (Pei and Grishin 2012) (Fig. 1).

Both FZD-CRD and FZ-CRD show homology with a conserved pattern of “*C*_*n*_*C*_*n*_*CX*_*8*_*CX*_*6*_*C*_*n*_*CX*_*3*_*CX*_*6,7*_*C*_*n*_*C*_*n*_*C*” (Pei and Grishin 2012) highlighted in Fig. 1b (*C*: conserved cysteine; *n*: a variable number of residues, *Xn*: n residues, and *Xn1, n2*: n1 to n2 residues) in α-helices forming a common Frizzled fold across four α-helices (Bazan and de Sauvage 2009). FZD-CRD has six residues between C7-C8 while receptor tyrosine kinases (RTKs) have seven residues for the same cysteine positions. The disulfide pattern between the cysteine residues of FZ-CRD is shown in Fig. 1b. The evolutionary conservation of the cysteine residues between these proteins might suggest structural importance of the disulfide bridges (Saldanha et al. 1998) and possibly CRD folding.

## Biological importance of FZ-CRD

### FZ-CRD interacts with Wnts and other ligands

FZD-CRDs control cell polarity and proliferation during embryonic development (Peifer 1999; Ye et al. 2010). Wnts and Wnt receptors interact with FZDs through their CRD initiating distinct downstream signalling pathways. For example, Wnt/Wg (Drosophila Wingless) ligands have been shown to bind to FZD-CRD with high affinity (Dann et al. 2001) and maintain tissue homeostasis (Ye et al. 2010). However, dysregulation of Wnt-FZD signalling results in many diseases and abnormalities (Wang et al. 2016) as deletion of FZD-CRD is shown to prevent Wnt/Wg binding (MacDonald and He 2012).

Our focus is on FZD4, MuSK and ROR2, which are considered as Wnt receptors. Nevertheless, it is important to note that FZ-CRD also binds to non-conventional Wnt ligands, such as the FZD4-Norrin interaction required throughout retinal vascular development (Ye et al. 2010; Ye et al. 2009; Smallwood et al. 2007). The FZ-CRD of FZD4 (FZ4-CRD) and the linker region has been shown to play a critical role in Norrin-FZD4 binding (Zhang et al. 2011; Bang et al. 2018), where a Norrin dimer interacts with two CRDs in a 2:2 stoichiometry (Chang et al. 2015), and the linker region found between the CRD and transmembrane domain (TMD) (Bang et al. 2018; Byrne et al., 2016), increases the affinity and binding of Norrin to FZD4 by 10 fold (Bang et al. 2018).

MuSK is a key player in synaptic differentiation, and acetylcholine receptor (AChR) clustering where postsynaptic differentiation is orchestrated by interactions of the proteoglycan agrin, low density lipoprotein receptor-related protein 4 (LRP4), docking protein 7 (DOK7) and receptor associated protein of the synapse (RAPSN). MuSK FZ-CRD is similar to Frizzled CRDs and interacts with Wnt4, Wnt11, and Wnt9a in vitro (Strochlic et al. 2012; Zhang et al. 2012).

ROR2 is important for embryonic development within the skeletal system and internal organs (Green et al. 2014). Interestingly, RORs share significant domain similarity to MuSK receptor (Yan et al. 2013; Bainbridge et al. 2014). ROR2 contains FZ-CRD which binds Wnt5a for activation (Ali et al. 2007). RTKs are activated by ligand-induced homo- and/or hetero-dimerization (Stroud and Wells 2004) and it has been proposed that Wnt5a activates Ror2 through dimerization via the FZ-CRD (Janda et al. 2012).

SMO FZ-CRD is homologous to Frizzled-CRD, and the former binds to the endogenous Wnt ligand and activates downstream Wnt signalling (Dann et al. 2001). FZ-CRD in SFRP (Bafico et al. 1999) and CPZ (Moeller et al. 2003) have been shown to bind Wnt and modulate the signalling pathway (Pei and Grishin 2012). The longest isoform of COL18A1 which contains FZ-CRD might be involved in intra-organ patterning during organ morphogenesis (Lin et al. 2001). Dysfunctional Wnt signaling causes various human diseases such as cancer, among many others.

## FZ-CRD folding is important for receptor function

**Fig. 2:**
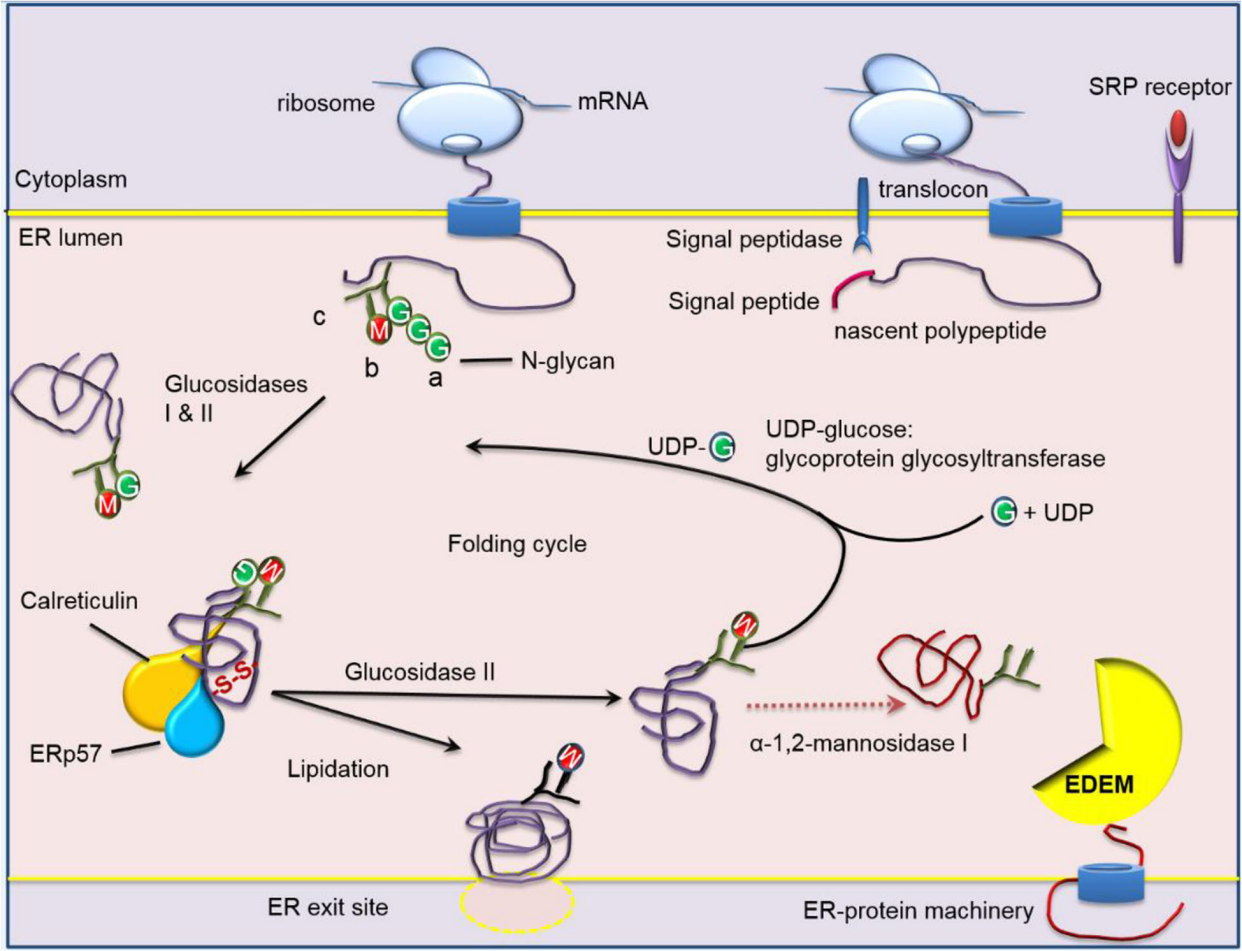
The glycoprotein folding cycle within the endoplasmic reticulum lumen. Protein glycosylation is a highly conserved process and plays crucial biological and physiological roles. Polypeptides translated on ribosomes from mRNA are escorted to an ER translocon via the signal recognition particle (SRP) and receptor. As the polypeptide enters the ER, an en bloc transfer N-glycans (Glc(3)Man(9)GlcNAc(2)) where glucose is represented as green circles and mannose as red, and N-acetylglucosamine (GlcNAc) is Y shaped green structure attached to the nascent polypeptide chain. FZD4 and MuSK have two N-glycosylation sites in their extracellular domains. α-glucosidase I and II (GI /GII) remove two of the three glucoses forming a monoglucosylated glycoprotein. This monoglucosylated protein is a signal for interacting with CNX and CRT, both lectins bound to protein disulfide isomerase family A member 3 (PDIA3). CRT is the soluble form of CNX and they form interchain disulfide bonds (S-S) with the bound glycoproteins. Removal of the last glucose by GII allows the glycoprotein to be released from the chaperones and leave the ER through ER exit sites to the golgi apparatus. Lipidation is a co or post-translational modification where lipid moieties are covalently attached to the polypeptide to increase hydrophobicity, conformation, and stability. Misfolded proteins trigger UDP-glucose-glucosyltransferase to re-add a single glucose on to the glycan and the cycle of protein folding is repeated. If the glycoprotein is permanently misfolded, the terminal mannose α1–2Man from the central arm of Man(9)GlcNAc(2), shown as a blue triangle, from the b branch of the oligosaccharide is removed by α-1,2-mannosidase I yielding a Man(8)GlcNAc(2) b-isomer. A second ER resident α-mannosidase I–like protein which lacks enzyme activity known as ER degradation-enhancing α-mannosidase I–like protein (EDEM), recognizes misfolded glycoproteins and targets them for ERAD machinery (Milhem 2015)

The endoplasmic reticulum (ER) serves as a central hub for efficient protein and lipid synthesis (Mandl et al. 2013). Glycosylation of polypeptides ensues on entry into the ER, and attached *N*-glycans moieties serve to support the structural and functional properties of glycoproteins on the cell membrane needed for key biological processes (Fig. 2).

Interestingly, the ER sustains a proper folding environment for FZ-CRD folding and activation of homo-and/or hetero-dimerization required for expression and function of the protein on the cell surface (Dann et al. 2001; Janda et al. 2012; Kaykas et al. 2004; Stiegler et al. 2009; Nile and Hannoush 2019). Among FZD members, FZD4 signalling and biological function is the most widely studied. Recently, structural deviations detected through I-TASSER structure predication server (Fredriksson et al. 2003) in FZ-CRD and seven transmembrane domain (TMD) of FZD4 are shown to be highly affected by mutations in FZ-CRD (Seemab et al. 2019). Seemab et al. show that FZD4 disease-causing missense mutations affect the K-S/T-XXX-W and T/S-X-V PDZ binding motifs resulting in major structural shifts within FZD4. FZ-CRD has been shown to be equally important for stabilization of the tertiary structure of the TMDs of FZDs (Yang et al. 2018a). Studies on recombinant FZ-CRD show an orderly folded domain which possesses both alpha-helices and beta-strands required for proper CRD folding (Roszmusz et al. 2001).

### Disruption in ER glycosylation results in ER protein misfolding

Polypeptides enter the ER via a translocon en route through the secretory pathway (Fig. 2). Challenges within the ER lumen milieu, affect the folding cascade of the polypeptide. Glycosylation of asparagine residues, N-glycosylation, is unique to the ER and marks the initiation of protein folding and is an essential protein modification (Helenius 1994) where a core unit made up of glucose (G): mannose (M) and N-acetylglucosamine (GlcNAc) (Glu(3)Man(9)GlcNAc(2)) with three branches (a, b and c) is transferred en bloc onto polypeptides in the rough ER lumen by the oligosaccharyltransferase enzyme (Kornfeld and Kornfeld 1985).

The a-branch or glucose-containing arm of N-linked glycans (Fig. 2) recruits molecular chaperones which assist efficient folding of glycoproteins (Helenius and Aebi 2004; Pearse and Hebert 2010). Therefore, the folding cycle is triggered by the removal of the terminal glucose residue (G) of the transferred triglucosylated glycan on branch a by α-glucosidase I (GI), a translocon associated protein. Trimming of the second glucose by an α-glucosidase II (GII), a luminal enzyme, supports co- or posttranslational association of folding polypeptides with the monoglucosylated glycan client (Glu(1)Man(9)GlcNAc(2) with the ER lectin chaperones membrane bound-calnexin (CNX) and its lumen soluble homolog-calreticulin (CRT). Both CNX and CRT (Helenius and Aebi 2004) in complex with the glycan directed oxidoreductase PDIA3, modulate disulfide bond formations within the monoglucosylated glycan promoting native three dimensional protein configurations.

UDP-glucose-glucosyltransferase (UGGT1) acts as a folding sensor and reglucosylates Man(7–9)GlcNAc(2) to restore the binding site for CXN and CRT to re-guide folding again (Fig. 2). Unfortunately, this cyclic quality control can be disrupted by the prolonged retention of the glycoprotein in the ER lumen and triggers the removal of mannose residues from the b and c branches of Man(9)GlcNAc(2) by ER mannosidases I and II form Man(7-8)GlcNAc(2), and consequently makes the glycoprotein unrecognizable by GII and UGGT1. The ER lectins of the ER degradation-enhancing alpha mannosidases-like protein (EDEM) family (EDEM1–3) act as mannosidases and recognize the mannose trimmed N-glycans which possess an energetically unstable conformation and these partially folded proteins are targeted for ERAD (Fig. 2). Following this close scrutiny, bona fide synthesized proteins may exit the ER to set off for their final destinations within the cell, or are secreted into the extracellular environment (Ahner and Brodsky 2004).

### ER-associated degradation

**Fig. 3:**
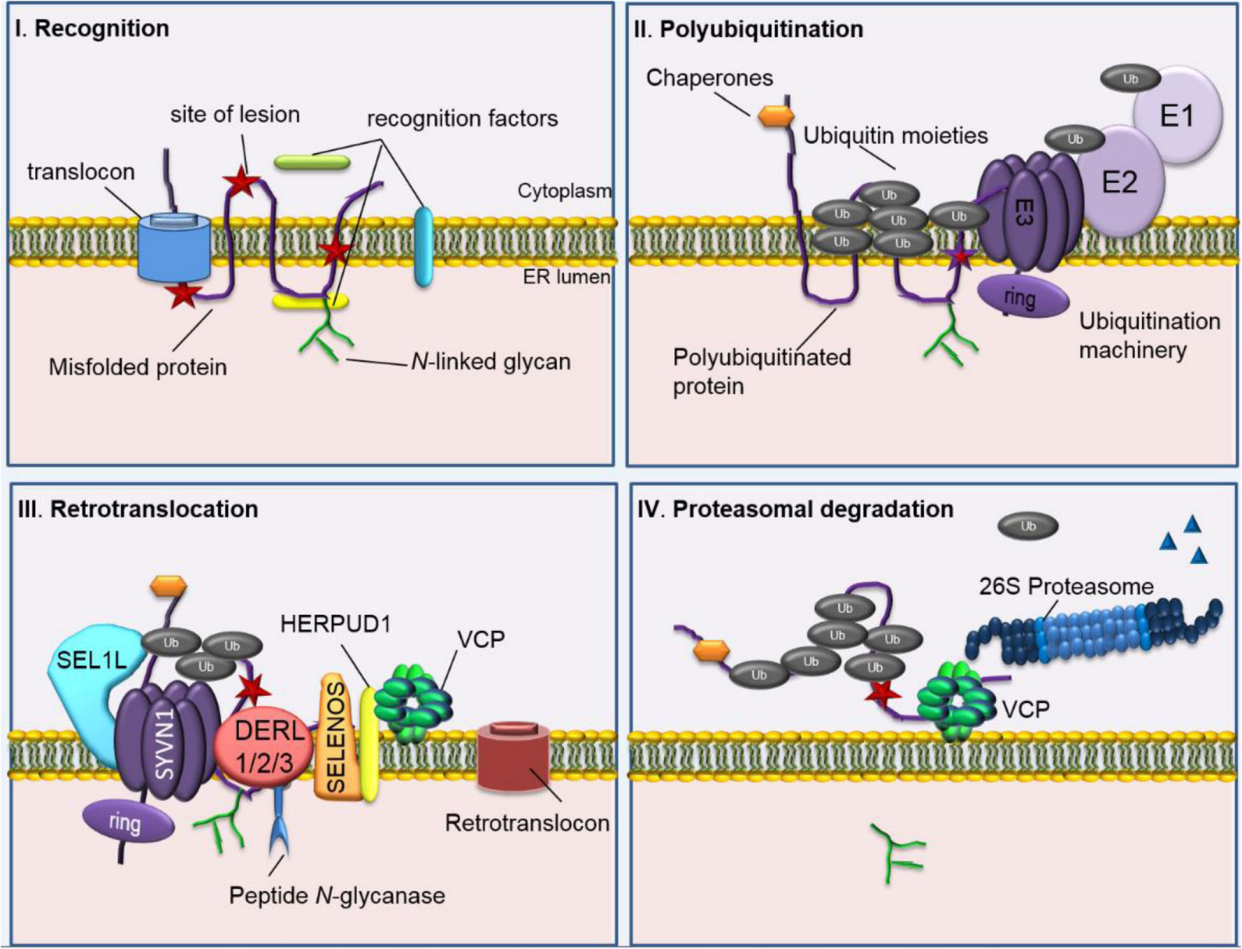
The four main steps for ERAD. *I*. *Recognition* occurs during protein synthesis. Here a misfolded region (red stars) are recognized by either cytoplasmic, ER luminal and/or transmembrane recognition factors depending on the site of lesion. *II. Polyubiquitination* starts when chaperones and co-chaperones direct the misfolded substrate to ubiquitination machinery. An ubiquitin activating enzyme (E1) transfers ubiquitin (Ub) (grey circles) to cysteine residue in an active site of an ubiquitin conjugating enzyme (E2) using ATP as energy. Ubiquitin ligase then transfers Ub to a lysine residue on the substrate protein. The latter process occurs on either the ER or cytoplasmic side of the membrane. *III*. *Retrotranslocation* ensues when the substrate protein is escorted to the dislocation machinery made up of a protein scaffold such as SEL1L adaptor subunit of ERAD E3 ubiquitin ligase (SEL1L), synoviolin 1 (SYVN1), cytochrome c oxidase assembly factor 7 (COA7) (not shown), derlin 1,2,3 (DERL1,2,3), selenoprotein S (SELENOS), homocysteine inducible ER protein with ubiquitin like domain 1 (HERPUD1), and valosin-containing protein (VCP). The substrate protein is removed either by passing through a retrotranslocon or by complete elimination of the protein. This is mainly done by the cytoplasmic ATPases associated with diverse cellular activities (AAA^+^ ATPase) p97 (commonly known as VCP), which interacts with Ub on the substrate and de-ubiquitinates the mutant protein and sends it off to the 26S proteasome. IV. *Degradation* is the final step where polyubiquitinated substrates are escorted to the 26S proteasome for degradation of faulty proteins. N-glycans are cleaved off by peptide N-glycanase associated with the ERAD machinery and Ub moieties are removed by de-ubuitinating enzymes found in the cytoplasm or in the proteasome cap to release small peptides shown as blue triangles (Milhem 2015)

Improper ER glycosylation, proteostasis, and fatty acid metabolism are linked to ER-associated degradation (ERAD) (To M et al. 2017) (Fig. 3), which clears misfolded proteins by mediating the ubiquitin (Ub)-dependent delivery of ER misfolded polypeptides to the 26S proteasome for proteolysis. Ubiquitination is a post-translational modification which serves to add Ub moieties to the substrate to allow for recognition as an ERAD substrate by the proteasome shown as step one in Fig. 3. Substrates are first monubiquitinated by E1, an ubiquitin-activating enzyme shown in Fig. 3, which transfers Ub via ATP (Adenosine triphosphate) to an active site cysteine (Schulman and Harper 2009) in E2, an ubiquitin-conjugating enzyme. Ubiquitin ligase (E3) acts as a platform for Ub moieties and then transfers ubiquitin from E2 to a lysine residue on the misfolded protein. Additional Ubs leads to the formation of polyubiquitin chains (PUCs) shown as step two in Fig. 3. Step three entails the movement of ERAD substrates from the ER to the cytoplasm for ubiquitination and proteasomal destruction by a process called retrotranslocation and degradation is the final step (step 4) where misfolded proteins are escorted by a 19S cap to the 26S proteasome. N-glycanase removes N-glycan residues and de-ubiquitinating enzymes remove Ub tags which then allow the proteasome core trypsin-like, chymotrypsin-like and caspase-like peptidases to cleave the misfolded protein into short peptides for recycling back into the cell.

ERAD clears the ER from faulty and toxic polypeptides and/or subunits of misfolded complexes (Pisoni and Molinari 2016), thus leading to more than 100 identified protein conformational diseases in humans (Aridor 2007; Guerriero and Brodsky 2012; Vembar and Brodsky 2008; Welch 2004; Needham et al. 2019).

### Importance of FZ-CRD in disease development

Little is known about the importance of FZ-CRD ER-folding in disease development. We previously hypothesized that FZ-CRD amino acid substitutions in FZD4, MuSK and ROR2 affect the tertiary structure of the polypeptide causing the respective proteins to malfold, traffic abnormally within the secretory pathway, consequently leading to loss-of-function of the receptors on the cell surface (Ali et al. 2007; Milhem et al. 2014). In the next section, we briefly discuss the effects of reported inherited pathogenic missense mutations on these receptors which we coin as “disorders of FZ-CRD”, shedding light on the importance of these mutations with ERAD as a common pathogenic cellular mechanism of disease.

## FZD4 inherited mutations cause familial exudative Vitreoretinopathy

Familial Exudative Vitreoretinopathy (FEVR; OMIM# 133780) is a hereditary condition where retinal blood vessels shows incomplete or no vascularization (Pendergast et al. 1998). Norrin/FZD4 proteins control the Wnt signaling pathway responsible for the regulation of endothelial growth and maturation throughout retinal vascular development (Ye et al. 2010; Ye et al. 2009; Smallwood et al. 2007). FEVR patients show variable phenotype expressions ranging from asymptomatic patients to an extreme level of complete blindness, and severe forms of FEVR in patients is observed when both alleles of the *FZD4* gene are mutated (Kondo et al. 2003).

FZD4 is a seven-pass transmembrane frizzled protein with an extracellular FZ-CRD (Fig. 1a) (Zhang et al. 2011; Seemab et al. 2019; Shen et al. 2015). FZDs also has a highly conserved YNXT motif found among all the paralogs of FZD family, and is located 5 residues after the CRD and is considered an N-glycosylation site important for Wnt-binding (Yan et al. 2013; Schwarz and Aebi 2011). FZD1–10, SFRP-3/4, ROR2, and CPZ contain similar N-glycosylation sites and therefore are able to bind Wnt (Yan et al. 2013).

**Fig. 4:**
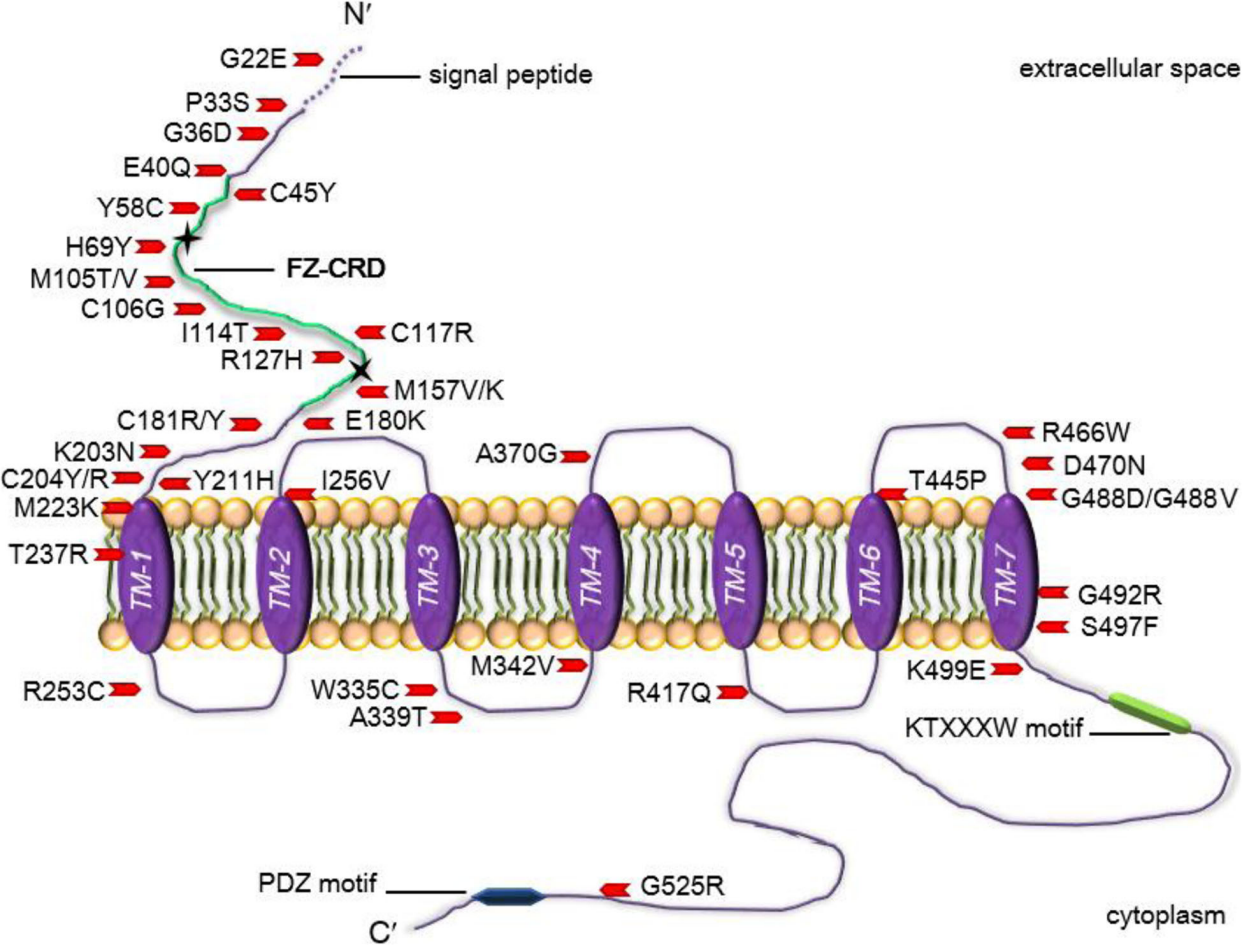
Schematic representation showing 40 reported FZD4 missense mutations dispersed across the protein and are associated with pathogenic FEVR. FZD4 contains a signal sequence at the amino (N′) terminus from amino acids 1 and 36/37; a conserved FZ-CRD region highlighted in green of approximately 122 amino acids in the extracellular domain containing a motif of 10 spaced cysteines between amino acid positions 40 through 161; a seven-pass TMD region labelled TM1–7 within amino acid positions 210 through 514; and a cytoplasmic domain with a KTXXXW motif found at amino acid positions 499 through 504, and a PDZ motif located close to the C′ terminal at amino acid positions 535 through 537. The two potential N-glycosylation sites are indicated by black stars at amino acid positions 59 and 144. Smallwood et al. have shown that the binding of Norrin to the CRD domain of FZD4 extends to include residue C204 (Smallwood et al. 2007). Amino acid positions and domains can be accessed from https://www.uniprot.org/uniprot/Q9ULV1

The first apo crystal structure of FZD4 has been recently published (Yang et al. 2018a). To date, 70 different FEVR pathogenic mutations have been reported for FZD4, of which 47 are missense mutations (Stenson et al. 2003). FZ4-CRD shows a cluster of missense mutations which cause FEVR (Kondo et al. 2003; Omoto et al. 2004; Jia et al. 2010) and their positions on the FZD4 protein is depicted in Fig. 4. Mutations which result in protein trafficking defects that do not conform to the scrutiny of the ER-quality control and are consequently disposed of by the proteasome are known as class II mutations. In the next section, we briefly highlight previous work carried out on FEVR causing missense mutations in FZ4-CRD (Milhem et al. 2014).

### FZD4 mutant proteins localize to the ER

The trafficking of 15 FZD4 missense mutations causing FEVR scattered throughout the protein were characterized for their N-glycosylation profiles. The Fz4-CRD is found within residues 42–167 and mutations surrounding FZ-CRD were also analyzed. The endo-β-N-acetylglucosaminidase H (Endo H) sensitivity in vitro assays showed immature proteins. FZD4 protein has two potential N-glycosylation sites in its extracellular domain and upon Endo H treatment, P33S (MacDonald et al. 2005), G36D (Toomes et al. 2004), H69Y (Omoto et al. 2004), M105V (Kondo et al. 2003) M105T (Toomes et al. 2004), C181R (Omoto et al. 2004), C204Y (Nikopoulos et al. 2010), C204R (Nallathambi et al. 2006), and G488D (close to the seventh TMD) (Kondo et al. 2007) mutants showed incomplete N-glycosylation, implying immature mutant proteins compared to wild-type FZD4 (WT-FZD4) which resisted Endo H treatment. C181R showed incomplete conversion by approximately 50%.

### Confocal fluorescence microscopy confirms ER localization

P33S, G36D, H69Y, M105T, C204R/Y and G488D FZD4 mutant showed a reticular pattern and co-localized within the ER during immunofluorescence confocal imaging. C204R/Y showed dual localization in the ER and on the plasma membrane (PM), and the amino acid substitutions at the 204 position result in the disruption of a vital cysteine disulphide bond which fails to bind Norrin (Smallwood et al. 2007; Zhang et al. 2011). Partial ER retention behaviour has previously been reported with mutations in cysteine residues (Rajan et al. 2009). M105V and C181R were shown to have a dual pattern of ER retention and PM expression which was seen by confocal fluorescence microscopy. However, when the M105V mutant was subjected to Endo H treatment, a single lower molecular-weight band was observed. A closer look at each mutation’s physicochemical properties and PolyPhen-2 (http://genetics.bwh.harvard.edu/pph2/ [in the public domain]) and SIFT (http://provean.jcvi.org/links.php [in the public domain]) is available in our study (Milhem et al. 2014).

### FZD4 mutant proteins are tagged with Ub moieties

As previously discussed, polyubiquitination is a pre-requisite for conjugates with multiple Ub moieties in the form of branched chains recognized by the proteasome. P33S, G36D, H69Y, M105T, C204R/Y and G488D FZD4 mutants were shown to be associated with Ub moieties to a much greater extent compared to WT with very-high-molecular-weight smears, suggesting polyubiquitination (Milhem et al. 2014). Once substrates are polyubiquitinated, they become exposed to the cytosol where they are recognized for early retrotranslocation. Therefore, the select FZD4 missense mutations are suggestive of tagging the FZ4-CRD mutant proteins for degradation by the ubiquitin/proteasome system (Fig. 3). Further to this, TOPflash reporter assay of FZ4-CRD mutations were recently shown to result in abnormal downstream signalling effects (Yang et al. 2018b) suggestive of their loss-of-function as proteins on the cell surface.

### Haploinsufficency of wild-type FZD4

FEVR displays autosomal dominant inheritance therefore, ER-trafficked mutants could possibly dimerize with the WT-FZD4 protein and trap it in the ER and hence cause a dominant negative effect. However, in our previous study, we show that the mutant proteins failed to retain the WT in the ER suggesting that the misfolded protein adopts conformations that inhibits dimerization or that the misfolded mutant is sequestered away from WT confirming haploinsufficiency of wild-type FZD4 in FEVR.

### Reducing temperature promotes folding and plasma membrane expression

Studies have shown that proteins that are kinetically stable and thermostable in the ER, but do not conform to a proper conformation, can still progress to the secretory pathway (Helenius and Aebi 2001). Incubating misfolded mutants at lower temperatures of around 27 °C, changes the kinetic and thermodynamic folding landscape of proteins and results in thermo-sensitive mutant proteins which informs about the possibility of therapeutic modulation of the protein. Therapeutic strategies in-place for class II proteins and their importance are discussed under “Current targeted strategies for conformational diseases”. Chemical chaperones (Denning et al. 1992) aid proper protein folding conformations, and support mutant protein PM expression.

**Fig. 5:**
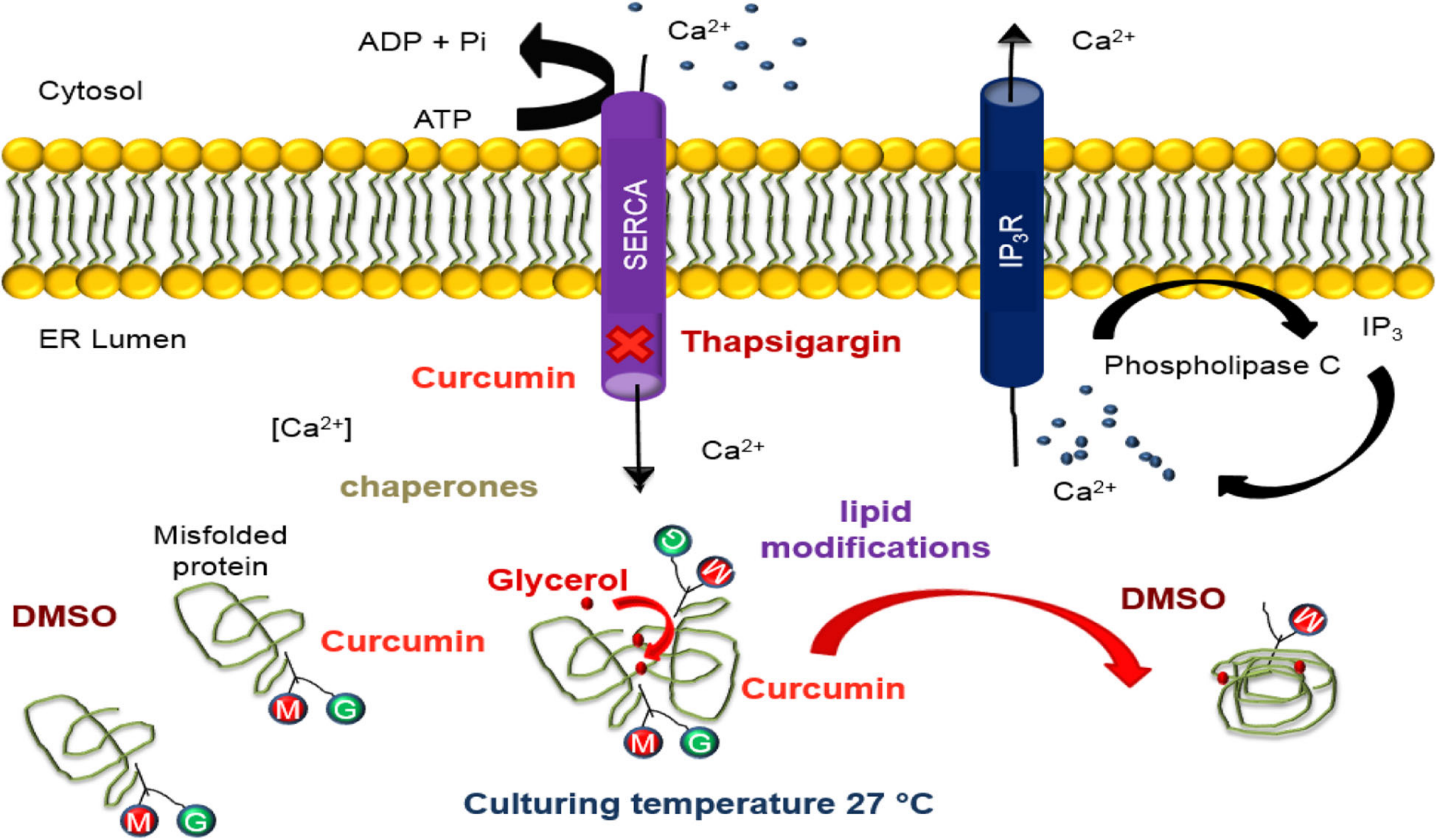
The effects of targeting the intracellular environment of proteostasis. Glycerol has the ability to increase the hydration layer of the protein and the intramolecular hydrophobic bonding strength. This in turn allows the free movement of proteins in the crowded environment of the ER thereby preventing aggregation of proteins. Differing concentrations (0.1–1%) of DMSO in a cell may increase protein synthesis of the misfolded proteins or by possibly overwhelming the quality control system. Thapsigargin acts as an inhibitor of the Ca^2+^ ATP2A pump pump and increases cytosolic calcium, and in doing so results in an enhanced rescue of mutant proteins (Robben et al. 2006). Curcumin is a nontoxic natural constituent of turmeric spice and affects the Ca^2+^ ATP2A pump found on the ER plasma membrane. Curcumin inhibits the pumps ability to maintain a high ER Ca^2+^ level which disturbs the ability of ER molecular chaperones to target the misfolded protein for ERAD, hence, allows the mutant protein to exit the ER. Post-translational modifications of lipid modifications and glycosylation can be therapeutically targeted to support disulfide bond and glycoprotein formation to enhance the proteostasis network (Milhem 2015)

Among these chemical chaperones are small synthetic chemicals such as glycerol, thapsigargin, dimethyl sulfoxide (DMSO), trimethylamine-N-oxide, calcium (Ca^2+^) pump inhibitors and curcumin among others (Fig. 5). Glycerol’s acts as an osmolyte, thereby increasing the hydration layer alongside the strength of the intramolecular hydrophobic bonding of proteins during folding, and in return prevents aggregation of mutant protein native conformations in the crowded milieu of the ER (Robben et al. 2006). DMSO (Zhang et al. 2003), trimethylamine-N-oxide (Song and Chuang 2001), calcium pump inhibitors (Egan et al. 2002) and curcumin (Egan et al. 2004) shift the folding equilibrium of mutant secretory proteins from an ER retention state towards a native state. DMSO’s solvation results in methyl groups which exposes a protein’s hydrophobic residues reducing aggregation. On the other hand, curcumin affects the internal cellular proteostasis within a cell, thereby enhancing favorable folding for proteins and consequently affecting its trafficking within a cell. Thapsigargin is a potent and selective inhibitor of the ubiquitous ATPase sarcoplasmic/endoplasmic reticulum Ca^2+^ transporting (ATP2A) found in mammalian cells. Thapsigargin which originates from plants increases cytosolic calcium, and in doing so, results in an enhanced rescue of mutant proteins from the ER (Fig. 5). M105T and C204Y were shown to be thermosensitive and were further exposed to chemical chaperones (Fig. 5) (Milhem et al. 2014). Our previous work showed that the immunofluorescence pattern of M105T and C204R mutants when cultured in the presence of 7.5% glycerol escaped from the ER by approximately 50 and 32%, respectively. This indicates that glycerol enhances the M105T and C204R mutants’ ER processing and allows the mutant proteins to exit the ER to the cell surface, albeit rather slowly compared to WT-FZD4. M105T and C204Y mutants were separately treated with 0.1% DMSO, 10 μM thapsigargin, 1 μM curcumin. The M105T mutant showed partial PM distribution by approximately 32% when treated with 0.1% DMSO and C204Y showed a lower pattern of rescue to the PM compared with M105T. Both M105T and C204Y showed no rescue at differing concentrations with either thapsigargin or curcumin. Interestingly, the immunofluorescence pattern of M105T and C204Y mutants showed trafficking from the ER to the PM when cultured with different chemical chaperones and therefore, FZD4 mutants might benefit from synergetic chemical chaperone treatment and/or other treatment strategies outlined in Table 1.

**Table 1:** Different treatment strategies currently in use for conformational disorders

Treatment Strategies	Description
Gene therapy	Gene therapy involves replacing the mutant copy of the gene with a wild-type functional protein.
Gene editing	CRISPR/Cas9 is a gene-editing strategy where only the mutated sequence of a mutant gene is edited and thereby corrected for proper function of the protein.
Gene correction in iPSCs	Using specialized induced pluripotent stem cells (iPSCs), CRISPR/Cas9 editing allows the correction of the gene within iPSCs increasing effectiveness of the technique.
Modulator Therapies--using differing mechanisms of action-	Modulators can be either potentiators, correctors [pharmacological chaperones & proteostasis regulators], stabilizers, or amplifiers.
	Modulators are pharmaceutical agents that targets specific defects in the mutant protein and/or modulate the intracellular environment. • Modulators target protein errors that occur post-transcriptionally, such as during protein folding, anterograde trafficking and further assist protein function and signaling following protein expression. ○ Correctors improve intracellular processing of misfolded proteins and increase plasma membrane expression. ○ Potentiators and stabilizers help the misfolded protein once expressed. Combination therapies with different mechanisms of actions, show greater efficacy. • Proteostasis regulators improve the overall quality of the proteostasis network within a cell. ○ Regulators can be designed to increase the function and availability of molecular chaperones, and consequently promote protein folding and/or reduce misfolding. ○ Regulators targeting the ER quality control can enhance the elimination of non-native conformations of polypeptides.
Stem cell therapy	Stem cell therapy is a tailored approach which is easy to proliferate and modify. It can further be coupled with CIRSPR/Cas9 and correct cells to a WT phenotype in the correct cell-line.
Antisense-oligonucleotide-mediated therapy	Single-stranded synthetic RNA-like molecules known as antisense oligonucleotides (ASOs) selectively change gene expression.
Non-viral vectors	Non-viral vectors have the ability to pack and deliver bulky DNA molecules with liposomal vectors.
mRNA-mediated therapy	Wild-type nucleotide sequence is targeted to the cell and has the ability to encode wild-type protein.
Proteasome inhibitors	Inhibition of proteasome, a unique proteolytic complex, prevents degradation of ubiquitinated proteins tagged for ERAD.

### Importance for FEVR patients

FEVR is a progressive disease which leads to blindness. M105V-FZD4 patients have been reported to show retinal folds, which eventually leads to retinal vascular tortuosity, alongside retinal degeneration (Jia et al. 2010). Patients with the M105T-FZD4 mutation have been diagnosed to show bilateral retinal detachment and partial and/or complete blindness at a young age (Toomes et al. 2004). Therefore, the phenotype of the M105V mutation is more severe than the M105T and this may be due to the slow trafficking of the M105V to the cell surface. The M105T is more debilitating and starts at a young age and our preliminary results show that M105T-FZD4 to be rescued to the cell-surface offering promise for FEVR patients with this genotype. Nonetheless, the exact effects of the studied mutants and synergetic chemical chaperone treatment on retinal vascularization and angiogenesis remain to be fully established.

## P344R-MuSK causes congenital Myasthenic syndrome

Congenital Myasthenic Syndromes (CMS, OMIM# 601296), is a group of genotypically and phenotypically heterogeneous group of neuromuscular disorders resulting in abnormal signal transmission and AChR clustering at the neuromuscular junction. Mutations in *MuSK* gene can cause CMS (Maselli et al. 2010; Chevessier et al. 2004; Mihaylova et al. 2009; Ben Ammar et al. 2013). MuSK protein is a 97 kilodalton type 1, single pass TK receptor with an extracellular ectodomain containing three immunoglobulin (Ig)-like domains and MuSK shares the same FZ-CRD homology as the FZD receptors (FZD1–8) (Stiegler et al. 2009; DeChiara et al. 1996; Masiakowski and Yancopoulos 1998). MuSK also harbors a transmembrane-spanning region, a juxtamembrane domain, a kinase domain and a C-terminal tail (Jennings et al. 1993) (Fig. 1a).

So far, six MuSK missense mutations have been reported in patients with CMS including D38E (Gallenmuller et al. 2014), P344R (Mihaylova et al. 2009), M605I, A727V (Chevessier et al. 2004), V790 M (Maselli et al. 2010) and M835 V (Ben Ammar et al. 2013). P344R-MuSK (Mihaylova et al. 2009) is found at the heart of the CRD (residues 314–409). Deletion studies of full-length MuSK lacking the FZ-CRD was expressed in MuSK−/− myotubes and results showed that MuSK FZ-CRD is required for AChR clustering (Zhou et al. 1999). The crystal structure of MuSK FZ-CRD is glycosylated and this contributes to a stabilized MuSK dimer (Stiegler et al. 2009) with the potential for MuSK oligomerization to elicit certain biological responses.

### P334R-MuSK mutant is underglycosylated and retained in the ER

Mislocalization was previously observed in three different cell lines, HeLa cells, COS-7 and HEK293, and a stable cell line was generated (Milhem et al. 2015). P344R-MuSK mutant was found to be predominantly localized to the ER as evidenced by its colocalization with ER-calnexin. This mislocalization away from the PM was further examined by its co-expression with enhanced green fluorescent protein-Harvey rat sarcoma viral oncogene homolog (EGFP-H-Ras) which localizes to the PM. The perinuclear and reticular distribution of the P344R-MuSK mutant is clearly distinct from that of EGFP H-Ras and from that of the wild type MuSK protein (WT-MuSK). MuSK has two potential N-glycosylation sites in its extracellular domain (Stiegler et al. 2009), one of which is within FZ-CRD (Till et al. 2002). PNGase (peptide-N (4)-(N-acetyl-beta-glucosaminyl) asparagine amidase) and Endo H sensitivity and resistance in vitro assays of the WT-MuSK and P344R-MuSK expressed proteins showed that P344R-MuSK is an under-glycosylated and immature protein (Milhem et al. 2015).

### P344R MuSK mutant is correctable by chemical chaperones and proteasome inhibitors

Interestingly, P344R-MuSK stable cell lines showed that P344R-MuSK is a thermosensitive protein and quantification of the immunofluorescence patterns under chemical chaperone treatment shows partial (~ 50%) rescue of the P344R mutant when treated with 2.5% glycerol*.* P344R-MuSK mutant was also separately treated with 0.1 and 1% DMSO, 10 μm thapsigargin or with 1 μM curcumin and showed partial plasma membrane re-distribution when treated with these chemical chaperones especially with 10 μM thapsigargin. Treatments showed enhanced P344R-MuSK protein processing compared to the untreated P344R-MuSK with quantification of western blots showing an increase in the stabilization of P344R-MuSK by approximately two-fold as compared to MuSK-WT under treatment (Milhem et al. 2015).

Proteasome inhibition with MG132 treatment caused the P344R-MuSK protein to increase two-folds compared to WT-MuSK. Further to this, P344R-MuSK showed around 50% of its in vitro autophosphorylation activity on stabilization. The pattern of multi-Ub-P344R-MuSK conjugates were reduced in MG132 treated samples and under chemical chaperone treatment (Milhem et al. 2015). Therefore, P344R-MuSK is a promising candidate for treatment strategies in place for class II conformational diseases.

### Treatment options for P344R-MuSK genotype patients

Patients harboring the P344R-MuSK genotype phenotypically show ptosis, fatigability on walking or exercise, incomplete ophthalmoparesis, bulbar weakness, with respiratory crises observed in certain patient cases. Acetylcholinesterase inhibitors combined with 3,4-diaminopyridine provided limited relief however, at times worsened symptoms (Mihaylova et al. 2009). Therefore, P344R-MuSK mutant is a good candidate for rescue from proteasomal degradation and will benefit from extending the investigation to a larger number of chemical chaperones or correctors (Needham et al. 2019). Our study highlights that prospective alternative personalized treatments for patients suffering from the P344R-MuSK mutation causing CMS, can be developed to target the source of the disease, rather than its consequences (Table 1).

## *ROR2* inherited mutations cause recessive Robinow syndrome

Robinow Syndrome is a skeletal dysplasia disorder which can be inherited as an autosomal dominant (DRS; OMIM 180700) or autosomal recessive (RRS; OMIM 268310) disorder (Robinow 1993). RRS results from loss-of-function of ROR2 from mutations in *ROR2* gene (Afzal et al. 2000). ROR2 is a glycoprotein and contains extracellular immunoglobulin like (Ig), FZ-CRD, and kringle domains. Missense mutations (C182Y, R184C, R189W, Y192D, R244W) and two reported double mutants (R344W-A245T) and (R189W-R366W) cluster in the FZ-CRD, and one (R366W) within the adjacent kringle domain, resulted in ER-trafficking and loss-of-function of ROR2 in patient samples (Ali et al. 2007; Chen et al. 2005).

## Other missense mutations reported in proteins with FZ-CRDs

SFRPs, CPZ, CORIN and COL18A1 also contain FZ-CRDs. Interestingly CORIN has an important role in mammalian cholesterol metabolism where CORIN binds and transports LDL to targeted cells via endocytosis. A reported a S472G missense mutation located within the first FZ-CRD of CORIN (Fig. 1a) was studied in preeclamptic patients and was found to be ER-retained due to misfolding (Dong et al. 2014). To our knowledge, missense pathogenic mutations in FZ-CRD of the other proteins have not been reported to result in ER-trafficking.

## Value of therapeutically targeting FZ-CRD ER-retained and misfolded proteins

### Current targeted strategies for conformational diseases

Current targeted strategies have been observed to account for the rescue of several different classes of misfolded and mislocalized proteins, and they are becoming increasingly important as therapeutics (Mohanraj et al. 2019). Over the last few years small molecules known as proteolysis targeting chimeras (PROTACs), were used to stimulate protein polyubiquitination and degradation (Lebraud and Heightman 2017). High throughput screening for molecules (Aymami et al. 2013; Tropak et al. 2007) and pharmacological chaperone therapy is in progress for genetic diseases (Aymami et al. 2013; Mohamed et al. 2017).

**Fig. 6:**
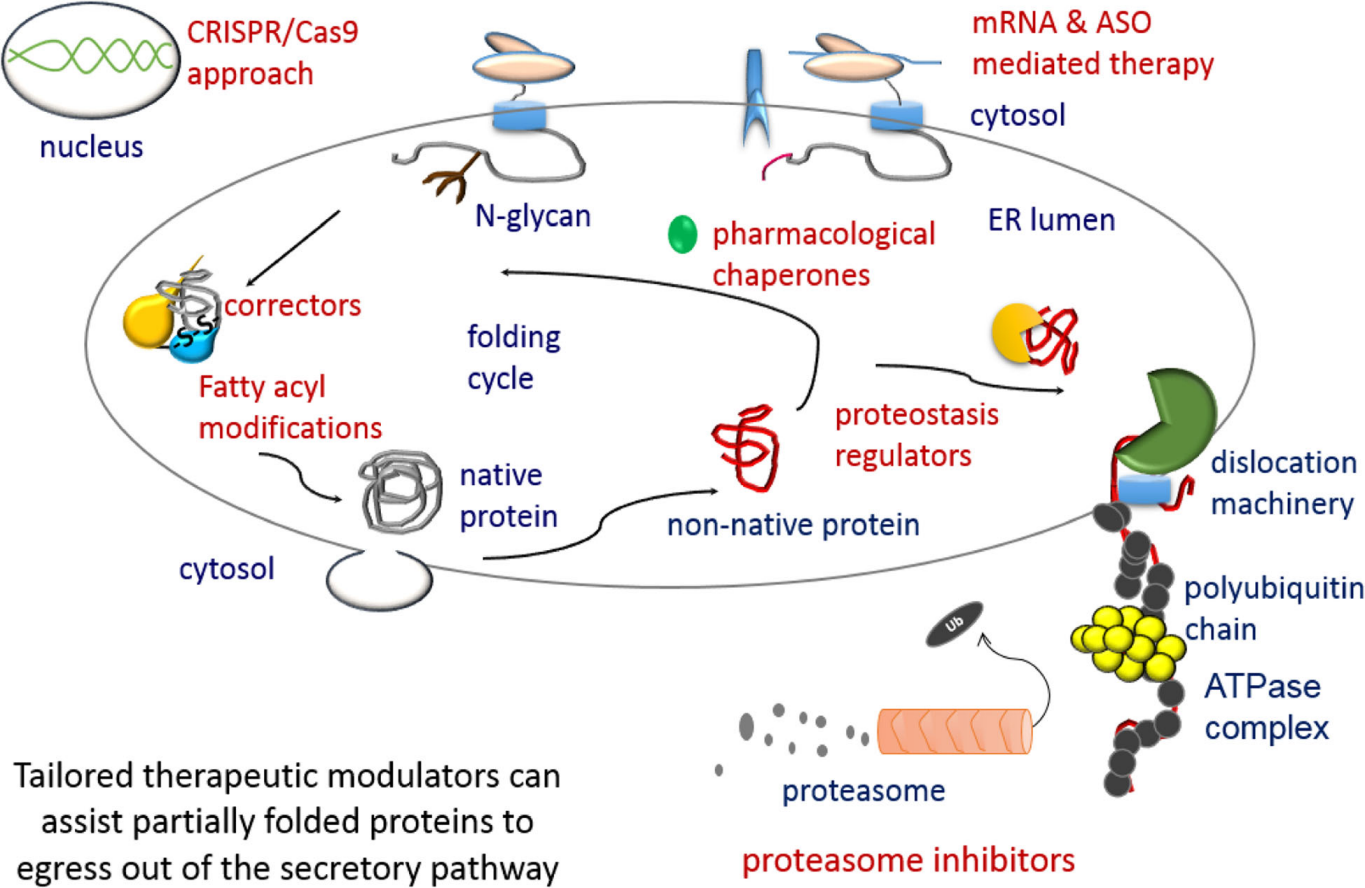
Summary: Therapeutically targeting the intracellular environment. The endoplasmic reticulum is a very important organelle for the proper folding of proteins that enter the secretory pathway. It contains stringent quality control checkpoints that monitor the folding of polypeptides and allow bone fide proteins to exit the ER and be expressed at their proper cellular localization. Research shows that proteins that are kinetically stable and thermostable in the ER, but do not conform to a proper conformation, can still progress to the secretory pathway and function similar to wild-type protein on the plasma membrane. Select therapeutic strategies are shown in red font. Pharmacological chaperones (correctors) can work at different levels of the folding cycle. Proteostasis regulators works with the ER quality control network and eliminate toxic non-native polypeptides. Fatty acyl modifications assist in proper cysteine bond formation and compact polypeptide folding. Abbreviations; ASO: antisense oligonucleotides, CRISPR/Cas9: clustered regulatory interspaced short palindromic repeats)/cas9 systems

Modulators show promising clinical efficacy and tolerability in different disease-causing proteins (Gamez et al. 2018). To name a few: cystic fibrosis transmembrane conductance regulator (CFTR) (Brown et al. 1996), α1-antitrypsin (Burrows et al. 2000), human phenylalanine hydroxylase, aquaporin-2 (Tamarappoo and Verkman 1998), vasopressin V2 receptor (Robben et al. 2006), ATP-binding cassette transporter proteins (Gautherot et al. 2012), α-galactosidase A (Chapple et al. 2001), Fukutin protein (Tachikawa et al. 2012), the prion protein PrP (Tatzelt et al. 1996), tumor suppressor protein p53, viral oncogene protein pp60, and ubiquitin-activating enzyme E1 (Chaudhuri and Paul 2006), adenosine triphosphate (ATP)-binding cassette subfamily A member 3 (Kinting et al. 2018), peroxisomal ABCD1 protein (Morita et al. 2019), cytochrome c oxidase assembly factor 7 (Mohanraj et al. 2019), disease-associated RPE65 retinoid isomerase proteins (Li et al. 2014), podocin-encoding gene NPHS2 (Serrano-Perez et al. 2018), and P-glycoprotein (Loo and Clarke 1997) among many others. Recent approaches to drug design, target the “root” cause of the disease as shown in Table 1 plus Figs. 5 and 6 (Esposito et al. 2016; Maiuri et al. 2017).

### Lipidation of FZ-CRD proteins remains less characterized

Lipid moieties attached during ER lipidation affect protein function through subcellular localization switching between membranes, plus folding, stability, and signalling within the cell. Therefore, protein glycosylation and lipidation are collaborative processes indispensable for the fine tuning of protein folding which allows bone fide proteins to egress out of the ER (Fig. 2). On exit from the ER, polypeptides transit through the secretory pathway for further processing and bone fide proteins are expressed correctly in the cell. Lipidation of proteins is linked to cancer, neurological and metabolic diseases among many others (To M et al. 2017; Jiang et al. 2018; Chen et al. 2018). Lipidation is important for cellular energy homeostasis, and is beyond the scope of this review, however, its importance in disease, compartmental trafficking, and proteostasis remains less characterized.

### Importance of lipidation in Wnt ligands and receptors biological function

For example, Wnt fatty acylation regulates signal transduction and S-palmitoylation (S-acylation) is shown to be an important modification for synaptic transmission and GPCR protein signalling regulation (Chen et al. 2018). *S*-palmitoylation involves 16-carbon palmitate (or other fatty acids) which are attached to cysteine residues (Chen et al. 2018). S-palmitoylation is a reversible posttranslational modification involving the covelent attachment of palmitate to proteins by palmitoyl acyltransferases, the latter contains a conserved DHHC-CRD (Linder and Deschenes 2007).

S-palmitoylation enhances the hydrophobicity of proteins, allowing reversible association with membranes, protein-protein interactions, and subcellular trafficking. For example, CRD of cysteine-string protein (CSP), a DnaJ/Hsp40-family chaperone, shows palmitoylation on cysteine residues and functions to localize the protein on the plasma membrane. Unpalmitoylated CSP shows slow binding capacity and rapidly dissociation from membranes, and ER accumulation (Greaves et al. 2008).

Wnt serine (Ser) acylation is important in the anterograde secretory pathway and allows for high-affinity interactions between Wnt and FZD8-CRD through Ser residues (Janda et al. 2012). Wnt Ser acylation is required for intracellular trafficking and membrane-association to allow Wnts to exit the ER. Ser acylation is also important between Wnt and its cargo receptor Wnt ligand secretion mediator (WLS). Within the Golgi complex, WLS receptors bind Wnt (Banziger et al. 2006) and escort Wnt to the cell surface. Wnt Serine acylation further interacts with lipoproteins to assemble Wnts into secretory particles (Jiang et al. 2018). Wnt’s palmitoleic acid (PAM) moiety guides the movement of WLS to the Golgi apparatus (Willert et al. 2003).

Mutations in Wnt cysteine residues result in irreversible oxidation of the Wnt proteins which causes the protein complex to bury the lipid modified Ser-residue internally, reducing hydrophobicity of the protein (Hosseini et al. 2019). Mutations in Wnt fatty acyl modification proteins are shown to cause certain embryonic developmental abnormalities (Hosseini et al. 2019).

### Unique lipid binding groove for FZD

Structural information on Wnt/FZ-CRD interactions allows the appreciation of the structural and functional role of intracellular lipid-protein interactions. Wnt lipid group(s) are post-translational modifications conserved in Wnts and important for Wnt signalling (Willert et al. 2003). It has been proposed that the FZ-CRD fold is compact and when the core α-helices are relaxed, the configuration might allow binding of a sterol/lipid-like ligand such as the lipid-modified Wnt and Hedgehog signalling molecule family (Hh) ligands. Wnt and Hh are both lipid morphogens where Wnt is modified with palmitoylated residues, and Hh with cholesterol at its C′-terminal and palmitoyl groups on an N′-terminal cysteine residue. Wnt and Hh might possibly bind with the flap-free sterol binding FZ-CRD fold (Bazan and de Sauvage 2009).

Wnt lipid groups have also been shown to directly engage with FZ-CRD, as shown in the 3.25 Å structure of *Xenopus* Wnt8 interacting with mouse FZ8-CRD (FZ-CRD of FZD8) (Janda et al. 2012). FZ-CRD forms a hydrophobic lipid-binding groove for Wnt PAM moiety similar to a co-receptor. FZ2-CRD is shown to display Wnt PAM bound to the groove (Tao et al. 2016). Recent structural data show human FZ7-CRD_−_ in-association with a free 24-carbon fatty acid, and FZ5-CRD with PAM (Nile and Hannoush 2016).

The CRD groove’s amino acid sequence is conserved across all 10 members of the FZDs receptors (Yang et al. 2018b; Tao et al. 2016). On dimerization and at the dimer interface, two lipid binding grooves close in on each other creating a lipid-binding cavity resembling a U-shaped cleft (Hosseini et al. 2019). Lipidation can directly affect protein activity and free *cis*-unsaturated fatty acids act as ligands able to induce FZ-CRD dimerization and higher-order oligomerization (Hosseini et al. 2019; Nile and Hannoush 2016). FZ2-CRD binding site for the lipid co-receptor can accept exogenous and endogenous lipids and Wnt PAM in-vitro *(*Tao et al. 2016*)*.

The above insights show how lipid co-receptor modifications and CRD’s interactions are important for proper expression of FZDs on the cell surface. Mutations in the lipid-binding groove of FZ2-CRD is shown to deter glycosylation, proper folding, and results in ER trafficking of the receptor (Tao et al. 2016). It is therefore plausible to assume that all FZ-CRD containing proteins require proper lipid modifications to allow bone fide receptor expression, however, this remains to be experimentally determined.

### Filling the gaps for FZ-CRD folding

Nile and Hannoush show recent development of FZ-CRD interactions with fatty acids which allow FZ-CRD to attain multiple conformations on intracellular fatty acid binding. Fatty acyl modifications at the hydrophobic cavity stabilizes FZD receptors, demonstrating a link between fatty acyl modifications and FZ-CRD’s proper folding. From the GPCRs, FZD4 has the most hydrophilic pocket which results in a compact structure, therefore, therapeutic ligands tailored towards the hydrophobic cavity pose a challenge (Yang et al. 2018a). FZD4 has different mechanisms for recognition of ligands as a result of the different configurations for the ‘open-closed conformations’ of the binding pocket cavity. Different from GPCRs, FZD4 does not have an allosteric binding site in the cavity among helices II, VI, or VII. Therefore, FZD4 possibly has the ability to recognize different allosteric ligands. It has been recently proposed that FZD4 has novel ligand-recognition and activation mechanisms different from other GPCRs (Yang et al. 2018b).

Proof-of-concept studies show that targeting protein lipidation might serve as an effective therapeutic strategy with several lipidation-related enzymes as attractive drug targets (Chen et al. 2018). This may still be as starting point for therapy as it aims at treating and/or alleviating the root cause of the disease symptoms and not the consequences of the disease.

Efforts in understanding how the FZ-CRD folds as previously described with other proteins such as the low density lipoprotein receptor (Jansens et al. 2002) may be necessary to understand how chaperones can target this region and prevent misfolding (Fig. 5). It is plausible to research chaperones that target the specific cysteine residues, to preserve the pattern and support the structural disulfide bonds and cysteine residues, so as to prevent misfolding altogether.

It may be useful to adjust the reduction-oxidation potential within the ER to allow FZ-CRD cysteine residues to fold more efficiently. The addition of an oxidizing agent in the microsomes of dogs was able to enter the ER lumen and act as cysteine reducing and oxidizing agent for influenza hemagglutinin (HA) (Marquardt et al. 1993). Protein folding of HA was enhanced either by direct oxidation of disulfide bonds or by further assisting the oxidative enzymes such as the ER disulfide isomerase. The adaptive UPR could be targeted with therapeutic agents to increase the transcriptional activation of chaperones that may further support folding of the protein. It can also transiently increase protein synthesis.

Complete or partial siRNA of co-chaperone partners is a novel way to rescue the ER-retained proteins (Fig. 6 and Table 1). Proteomics is a growing field and has been used to assess CFTR protein interactions also known as the CFTR interactome. Wang et al. proposed the reason that ΔF508-CFTR failed to fold correctly was because the mutant was unable to fold under the dynamics set forth by the ER-chaperone folding environment known as the “chaperome”. The steady-state dynamics of the ER folding machinery were not in its favor. As a consequence, the Hsp90 co-chaperone which was shown to interact with CFTR’s protein folding in the ER was silenced to restore the energetically favorable environment for this mutant (Wang et al. 2006). Mapping of the CFTR interactome and modifier genes is invaluable for understanding the proteomics network (Lim et al. 2017).

It is important to understand further the genotype-phenotype correlation and disease pathogenesis. Our post-genomic era, has seen an unparalleled evolution of genomic manipulation, plus the regulation of gene expression. Genome editing such as TALEN (Transcription Activator-Like Effector Nucleases) (Mariano et al. 2014; Peng et al. 2014; Nemudryi et al. 2014; Schiml et al. 2014) and CRISPR (Clustered Regulatory Interspaced Short Palindromic Repeats)/Cas9 systems (Mariano et al. 2014; Nemudryi et al. 2014; Schiml et al. 2014) are reliable tools for genome engineering (Table 1). They are useful for cell-based human hereditary disease modeling (Nemudryi et al. 2014). Here one could make a cell based model with the desired mutation incorporated in the appropriate cell lines. This would allow the visualization of the cellular processes such as chemical chaperone treatment and small interfering RNA (siRNA) knockdown of ERAD components.

Interestingly, antibodies selectively targeted FZDs in preclinical models of breast, colon and liver cancer (Pode-Shakked et al. 2011; Wei et al. 2011). Wnt signalling has been altered in pre-clinical models by improved drug-discovery platforms (Anastas and Moon 2013), thus paving the way for the discovery of more effective and tailored drugs.

## Discussion and future outlook

To finish the details of FZ-CRD folding, the next step would is a major challenge as it would entail understanding the role of protein energetics, lipidation, and chaperone functions which are indispensable in the folding of cargo for ER export. All represent attractive coupled therapeutic targets for conformational diseases, including cystic fibrosis and neurodegenerative disorders (Fig. 6).

Testing of the FEVR-causing mutations in FZD4 deficient retinal cell lines and the P344R-MuSK in MuSK deficient myotubules are necessary to further understand how each mutant interacts in its own cellular milieu; to appreciate both the folding of FZ-CRD in the presence of the mutant and observe the stability of the mutant FZ-CRD with therapeutic targets. With the aid of current computer–based stimulations and bioinformatic tools, this is feasible.

The importance of the proteins’ interplay with chaperones in the ER is vital for siRNA knockdown as a target for therapy. It is important to further study interacting partners and whether these mutants interact with general chaperones, such as heat shock protein family A (Hsp70) member 5 (HSPA5), or specific chaperones such as the CFTR’s Aha chaperone and the Boca chaperone specific for LRP5/6 homologue arrow in *Drosophila melanogaster*; needed for WLS signal transduction (Culi and Mann 2003). Targeting DNA, RNA, or polypeptides in disease, is an emerging field, which is growing fast as insights into disease causation and small molecules is made available (Angelbello et al. 2018).

Alleviating the pathogenesis disorders of FZ-CRD will require novel emerging therapeutics, such as, structure-guided drug discovery (Winter et al. 2012), and/or the arrangement and regulation of fatty acids alongside FZ-CRD intracellular interactions. Targeting and/or modulating FZ-CRD could potentially aid disease biology. FZ-CRD structural data has shed light on novel players within the therapeutic landscape of conformational diseases. This could further pave the way to look within the cell at lipidation in other conformational disorders, resulting in a newly explored paradigm to promote folding of conformational diseases.

## Conclusion

Insights from this review show a hotspot for the clustering of missense mutations in FZ-CRD resulting in different misfolded proteins, each responsible for eliciting different debilitating diseased states. FZ-CRD can be manipulated with chemical chaperones and therefore is possibly amenable to therapy providing proof-of-principle that conformational disorders can be corrected. Recent work highlights that lipidation of class II proteins is vital for proper folding of proteins and anterograde trafficking. Elucidating the cellular and molecular mechanisms of disease serves as a platform to further understand the proteostasis network, protein-lipid interactions and appreciate the complexity of structural data. Therefore, from this review, it is envisioned that more molecular players in the FZD4, MuSK and ROR2 pathway will be discovered and the folding of FZ-CRD will be sought after to design small molecules for improving expression and stability of the mutant proteins at the cell surface.

Our review further opens the door for looking at other conserved modules that cluster mutations across other conformational diseases. It could also possibly lay the foundations for discovering alternative therapeutic measures for FEVR, CMS and RRS patients, the novelty of which is mutation specific which makes it personalized or alternatively targets disorders of FZ-CRD.

## Data Availability

Raw data, materials and methods can be assessed at: (Ali et al. 2007; Milhem et al. 2014; Milhem et al. 2015; Chen et al. 2005; Milhem 2015).

## Abbreviations

AAA ATPase: ATPases Associated with diverse cellular Activities
ABCC1: ATP binding cassette subfamily C member 1
ATP: Adenosine triphosphate
ATP2A: ATPase sarcoplasmic/endoplasmic reticulum Ca^2+^ transporting
C: Cysteine
C′: Carboxyl terminal
Ca^2+^: Calcium
CFTR: Cystic fibrosis conductance regulator
CMS: Congenital myasthenic syndrome
COL18A1: Collagen type XVIII alpha 1 chain
CORIN: Corin, serine peptidase
CPZ: Carboxypeptidase
CRD: Cysteine-rich-domain
CRISPR: Clustered regulatory interspaced short palindromic repeats)/cas9 systems
CRT: Calreticulin
CSP: Cysteine-string protein
CXN: Calnexin
DERL1/2/3: Derlin 1/2/3
DMSO: Dimethylsulfoxide
DOK7: Docking protein 7
E1: Ubiquitin activating enzyme
E2: Ubiquitin conjugating enzyme
E3: Ubiquitin ligase enzyme
EDEM: ER degradation-enhancing α-mannosidase I–like protein
EGFP *HRAS*: Enhanced green fluorescent protein-Harvey rat sarcoma viral oncogene homolog
Endo H: Endo-β-N-acetylglucosaminidase H
ER: Endoplasmic reticulum
ERAD: Endoplasmic reticulum associated degradation
FEVR: Familial exudative vitreoretinopathy
FZ4-CRD: Frizzled class receptor 4 cysteine-rich-domain
FZ-CRD: Frizzled-like cysteine–rich domain
FZD: Frizzled receptors
FZD4: Frizzled class receptor 4/frizzled family receptor 4
FZD-CRD: Frizzled cysteine–rich domain
GAPDH: Glyceraldehyde-3-phosphate dehydrogenase
GI/GII: α-glucosidase I and II
GlcNAc: N-acetylglucosamine
GPCRs: G-protein-coupled receptors
HA: Hemagglutinin
HEK293: Human embryonic kidney cell Line 293
HeLa: Henrietta Lacks (human epithelial adenocarcinoma cell line)
HERPUD1: Homocysteine inducible ER protein with ubiquitin like domain 1
Hh: Hedgehog
HSPA5/BiP: Heat shock protein family A (Hsp70) member 5
LDLR: Low density lipoprotein receptor
LRP: Low density lipoprotein receptor related protein
mRNA: Messenger ribonucleic acid
MuSK: Muscle, skeletal, receptor tyrosine kinase
N′: amino terminal
N-glycan: Glu(3)Man(9)GlcNAc(2)
PAM: Palmitoleic acid
PDIA3: Protein disulfide isomerase family A member 3
PM: Plasma membrane
PNGase F: Peptide-N(4)-(N-acetyl-beta-glucosaminyl) asparagine amidase
PUCs: Polyubiquitin chains
RAPSN: Receptor associated protein of the synapse
RNA: Ribonucleic acid
ROR2: Receptor tyrosine kinase-like orphan receptor 2
RS: Robinow syndrome
RTKs: Receptor tyrosine kinase
SEL1L: SEL1L adaptor subunit of ERAD E3 ubiquitin ligase
SELENOS: Selenoprotein S
Ser: Serine
SFRP: Secreted frizzled-related protein
siRNA: Small interfering RNA
SMO: Smoothened, frizzled class receptor
SP: Signal peptide
SRP: Signal recognition particle
SYVN1: Synoviolin 1
TALEN: Transcription activator-like effector nucleases
TMD: Transmembrane domain
Ub: Ubiquitin
UPR: Unfolded protein response
VCP: Valosin containing protein
WLS: Wnt ligand secretion mediator
Wnt: Wnt family/Wingless-type MMTV integration site family
WT: Wild-type
ΔF508 CFTR: Deletion of phenylalanine (F) at position 508 in the CFTR protein
